# Genetic and Epigenetic Alterations of *Brassica nigra* Introgression Lines from Somatic Hybridization: A Resource for Cauliflower Improvement

**DOI:** 10.3389/fpls.2016.01258

**Published:** 2016-08-30

**Authors:** Gui-xiang Wang, Jing Lv, Jie Zhang, Shuo Han, Mei Zong, Ning Guo, Xing-ying Zeng, Yue-yun Zhang, You-ping Wang, Fan Liu

**Affiliations:** ^1^Beijing Vegetable Research Center, Beijing Academy of Agriculture and Forestry Sciences, Key Laboratory of Biology and Genetic Improvement of Horticultural Crops (North China), Ministry of AgricultureBeijing, China; ^2^Yangzhou UniversityYangzhou, China; ^3^Zhalute No.1 High SchoolTongliao, China

**Keywords:** introgression lines, genetic diversity, *Brassica oleracea*, *Brassica nigra*, somatic hybridization, epigenetic variation, cauliflower

## Abstract

Broad phenotypic variations were obtained previously in derivatives from the asymmetric somatic hybridization of cauliflower “Korso” (*Brassica oleracea* var. *botrytis*, 2*n* = 18, CC genome) and black mustard “G1/1” (*Brassica nigra*, 2*n* = 16, BB genome). However, the mechanisms underlying these variations were unknown. In this study, 28 putative introgression lines (ILs) were pre-selected according to a series of morphological (leaf shape and color, plant height and branching, curd features, and flower traits) and physiological (black rot/club root resistance) characters. Multi-color fluorescence *in situ* hybridization revealed that these plants contained 18 chromosomes derived from “Korso.” Molecular marker (65 simple sequence repeats and 77 amplified fragment length polymorphisms) analysis identified the presence of “G1/1” DNA segments (average 7.5%). Additionally, DNA profiling revealed many genetic and epigenetic differences among the ILs, including sequence alterations, deletions, and variation in patterns of cytosine methylation. The frequency of fragments lost (5.1%) was higher than presence of novel bands (1.4%), and the presence of fragments specific to *Brassica carinata* (BBCC 2*n* = 34) were common (average 15.5%). Methylation-sensitive amplified polymorphism analysis indicated that methylation changes were common and that hypermethylation (12.4%) was more frequent than hypomethylation (4.8%). Our results suggested that asymmetric somatic hybridization and alien DNA introgression induced genetic and epigenetic alterations. Thus, these ILs represent an important, novel germplasm resource for cauliflower improvement that can be mined for diverse traits of interest to breeders and researchers.

## Introduction

Cauliflower (*Brassica oleracea* var. *botrytis*, 2*n* = 18, CC genome) is a major vegetable crop valued worldwide for its nutrition and flavor. As with other modern crop species, intense selection for preferred traits, and a tendency for inbreeding have resulted in low genetic diversity among cauliflower breeding resources. To address this problem, related species such as black mustard (*Brassica nigra*, 2*n* = 16, BB genome), with a large reservoir of genes conferring desirable characteristics, have been proposed as a valuable source of genetic diversity for *Brassica* crop improvement. In principle, this diversity can be transferred into crops via sexual hybridization and subsequent backcrossing. However, in practice, genetic manipulation using distantly related plants has been severely restricted by difficulties in creating the initial sexual hybrid and by sterility issues in the early backcross generations (Glimelius, 1999; Jauhar, 2006). Asymmetric somatic hybridization is a potential alternative for gene transfer from relatives to cultivated crops, especially when wide crosses are not applicable (Gerdemann et al., 1994; Xia, 2009). The Brassicaceae is a model plant family commonly used for somatic hybridization (Glimelius, 1999; Navrátilová, 2004), resulting in interspecies or even intertribal hybrids. Moreover, agronomically important traits, such as disease resistance and specific fatty acid compositions, have been successfully integrated into the crops (Gerdemann et al., 1994; Hansen and Earle, 1994, 1995, 1997; Wang et al., 2003; Tu et al., 2008; Scholze et al., 2010).

*Brassica* is also an excellent model for the study of allotetraploid speciation. *Brassica* is closely related to the classic plant model *Arabidopsis* and a wealth of germplasm exists from species in this genus. Three diploid species are widely cultivated: *Brassica rapa* (AA, *x* = 10), *B. nigra* (BB, *x* = 8), and *B. oleracea* (CC, *x* = 9). Recent interspecific hybridization among these species has resulted in polyploidization and the production of three allotetraploid species: *Brassica napus* (AACC, *x* = 19), *Brassica juncea* (AABB, *x* = 18), and *Brassica carinata* (BBCC, *x* = 17) (Nagaharu, 1935).

The formation of allotetraploids is an influential mode of speciation in flowering plants, often accompanied by rapid, extensive genomic and epigenetic changes that globally alter gene expression, termed “genomic shock.” These include fragment gain and loss through chromosome rearrangement or the activation of transposable elements (Kashkush et al., 2003; Kraitshtein et al., 2010), extensive alteration of DNA cytosine methylation (Zhao et al., 2011; Gautam et al., 2016), histone modification (Ha et al., 2011), and changes to small RNA (Ha et al., 2009). However, genomic-shock-induced changes during sexual polyploid synthesis should be distinct from the changes that occur from genomic shock during somatic hybridization. Somatic hybrids combine both the nuclear and cytoplasmic genomes within a single cell. Therefore, the introgression of chromatin segments via asymmetric somatic hybridization likely occurs via non-homologous end-joining of fragmented genome pieces, rather than by the homologous recombination that occurs through sexual reproduction (Liu et al., 2015). Moreover, the epigenetic states of somatic cells and gametal cells tend to differ, given that gametal cells are more conserved to ensure genetic fidelity (Bird, 1997, 2002; Liu et al., 2015). Thus, the variations induced by “somatic genomic shock” likely have unique genetic and epigenetic characteristics compared with “sexual genomic shock.” However, little data exist on the exact nature of these differences, or if the types of genomic shock indeed differ in their effects.

A number of hybrid progenies have been regenerated from asymmetric somatic hybrids between cauliflower “Korso” and black mustard “G1/1.” The hetero-cytoplasmic nature of these hybrids was confirmed through the finding that most progenies have chloroplast genomic components from “G1/1” and mitochondrial DNA from “Korso,” while mitochondrial genome recombination occurred in a few hybrids (Wang G. X. et al., 2011). Among the progenies, dozens of lines containing 18 chromosomes showed some obvious characters of “G1/1” origin, such as waxless leaves with lobes and ears, as well as green petioles; however, they were also densely covered with trichomes and exhibited purple stems and leaf veins, characteristic of “Korso.” This combination of characters indicated that they were putative introgression lines (ILs). Although hybrid synthesis primarily focused on the transfer of disease-resistance genes from “G1/1” into cultivated cauliflower (Wang G. X. et al., 2011) because the former is resistant to several *Brassica* pathogens (Westman et al., 1999), the putative ILs contained considerable genetic diversity for many traits beyond disease resistance. Therefore, these lines should possess many characters that differ from near-isogenic lines and feature discrete portions of “G1/1” chromatin in a “Korso” genetic background. Additionally, asymmetric somatic introgression should induce further diverse genomic variations involving change in DNA sequences and epigenetic modifications. To verify this, we used 12 putative ILs shown to be phenotypically stable for several generations to characterize genetic and epigenetic alterations from “somatic genomic shock.”

## Materials and methods

### Plant materials

**Figure 1:**
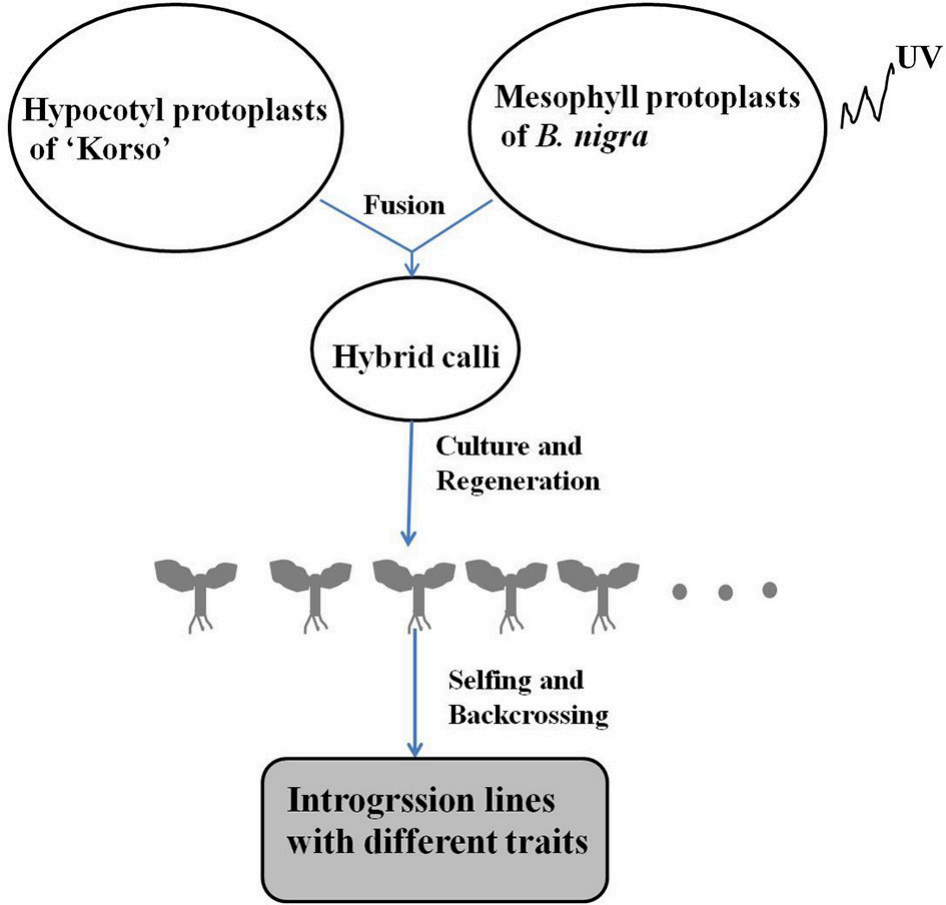
**Generation of introgression lines via somatic hybridization**.

Asymmetric somatic hybridization between cauliflower “Korso” and black mustard “G1/1” resulted in the establishment of 117 individual hybrids in the field; of these, 13 fertile plants with preferred traits were selected for continued selfing and backcrossing (Wang G. X. et al., 2011). Hundreds of derivatives were obtained following year-by-year selection (since 2006) that combined phenotypic observation, cytological and molecular analysis, as well as pathogen-resistance assays (Figure 1). In this study, 28 putative ILs were chosen for phenotypic diversity and chromosomal constitution analysis, based on possession of cauliflower-like morphology and phenotypic stability over three generations. Next, 12 putative ILs (IL1-12) were used for genetic an epigenetic analysis. Chinese cabbage “Asko” (*B. rapa*, AA, 2*n* = 20) and tetraploid *B. carinata* (BBCC, 2*n* = 34) were chosen as reference materials for molecular and chromosomal analysis. The selfed seeds from ILs, parents, and *B. carinata* were sown in a greenhouse and are available upon request.

### Multi-color FISH analysis

Multi-color FISH was conducted on meiosis metaphase I chromosomes of parent lines and putative ILs to confirm their chromosomal composition. *B. nigra* and CentBr (centromeric-specific tandem repeats of *Brassica*; accession numbers: CW978699 and CW978837, respectively; Wang G. et al., 2011) DNA were selected as probes and labeled with biotin (dig)-14-dUTP (Roche, Indianapolis, IN) using nick translation with an average length of 500 bp. Slides were prepared following a previously published protocol (Zhong et al., 1996) with minor modifications. To decompose the cell walls of pollen mother cells, anthers approximately 1–3 mm in length were digested at 37°C for 3 h in an enzyme mixture containing 6% cellulose R-10 and 6.5% pectinase (Sigma, solution in 40% glycerol). Previously described methods (Wang G. et al., 2011) were followed for FISH analysis. Hybridized probes were visualized using an FITC-conjugated avidin antibody or rhodamine-conjugated anti-digoxin antibody (Roche, Indianapolis, IN). Chromosomes were counterstained using 0.1 mg/mL DAPI (Vector Laboratories, Burlingame, CA). Images of the signals and chromosomes were captured using a CCD camera (QIMAGING, RETIGA-SRV, FAST1394) attached to a Nikon Eclipse 80i epifluorescence microscope (Tokyo, Japan). Image contrast and brightness were adjusted in Adobe Photoshop (8.0).

### SSR, AFLP, and MSAP fingerprinting

Simple sequences repeat (SSR), amplified fragment length polymorphism (AFLP), and methylation-sensitive amplification polymorphism (MSAP) fingerprinting were performed following previously described methods (Shaked et al., 2001; Liu et al., 2015). Sixty-five SSR, 77 AFLP, and 16 MSAP primer combinations were used; for SSR and AFLP, markers were collected from the A genome of *B. rapa* due to the limited number of cytological and genetic studies on the B genome of *B. nigra* (Tables S1–S3). For AFLP and MSAP, two independent technical replicates were performed for each of the three biological replicates. Only clear and reproducible bands larger than 100 bp were scored.

## Results

### Phenotypic diversity of introgression lines

**Figure 2:**
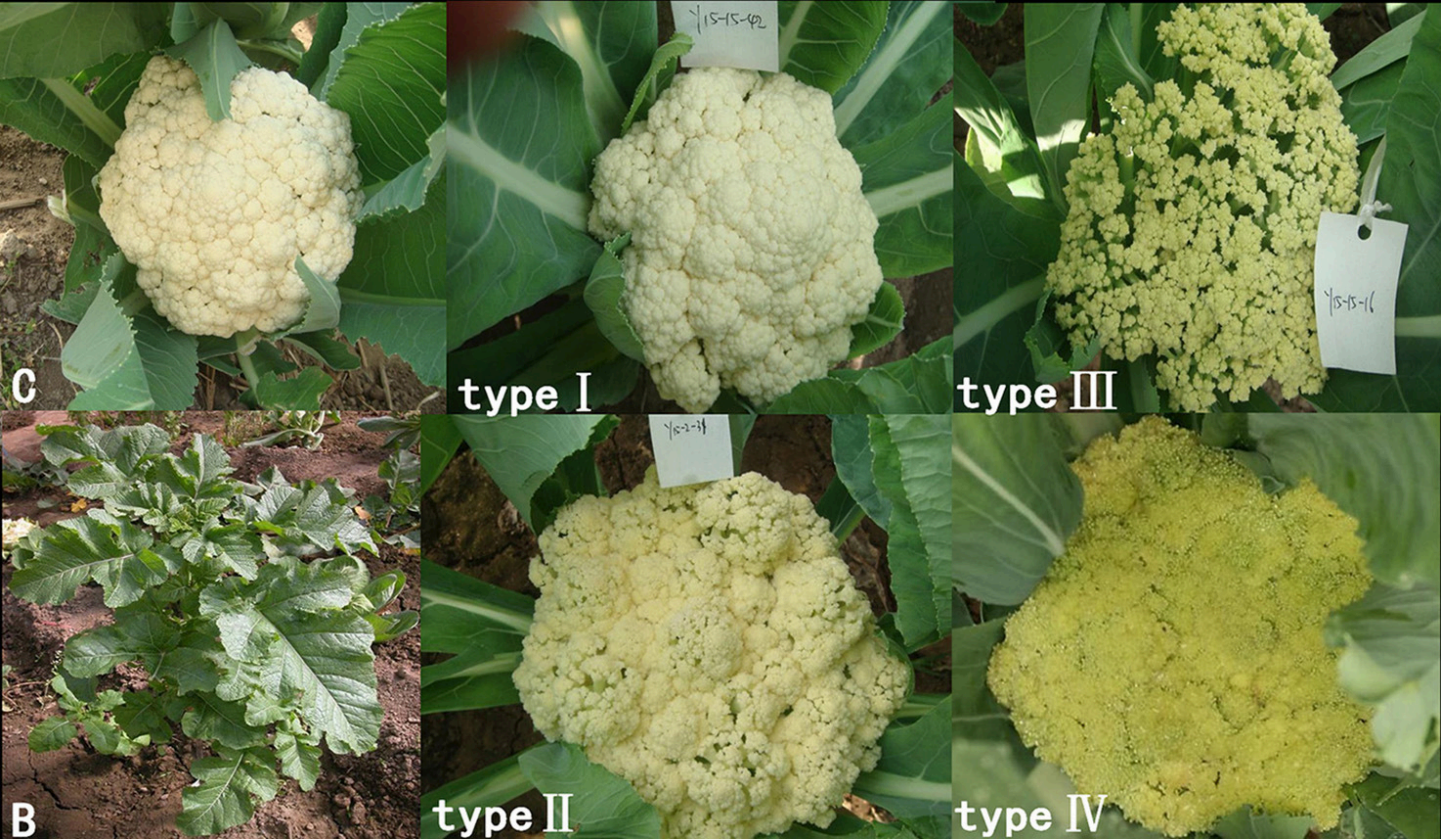
**Four types (I–IV) of representative flower heads in introgression lines**. C: “Korso,” B: “G1/1.”

The 28 ILs were extremely diverse in multiple morphological and physiological traits (Table 1), including distinct variations in leaf or flower morphology, fertility, and resistance to pathogens (i.e., black rot or clubroot). Here we describe diagnostic differences in flower heads as an example. Four phenotypic groups were apparent based on flower curd characters (Figure 2). In the first group (type I), flower heads were white, compact, and hemispheric, with a wheel-like radial arrangement; flower buds were small, granular, and close. The second group (type II) possessed flat, loose flower heads with no obvious wheel-like arrangement; flower buds were light yellow, tiny, and soft, while the pedicel was green and slightly longer than Type I pedicels. The third group (type III) possessed relatively flat flower heads and pale yellow-green color; flower buds were small, loose, soft, and distributed in clusters, but with no obvious ball flower formation. The pedicel was green and long. The flower heads of the fourth group (type IV) were relatively flat, yellow green, with small flower buds that were fine and soft.

**Table 1:** **Phenotypic traits associated with introgression lines (IL1-12) and their parents**.

**Materials**	**Leaf traits**	**Curd traits**	**Other special traits**	**Curd morphology**
“Korso”	Ovate; shallowly serrated margins; lacking auricles; waxy, glossy, and hairless surface	White and firm curd; short pedicel	Susceptible to both black rot *Xcc*15 and clubroot race 4; potassium content: 156 mg/100 g; phosphorus content: 36 mg/100 g	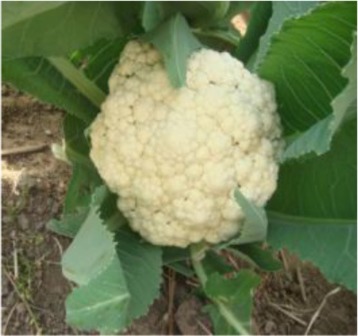
“G1/1”	Almost triangular; possesses auricles; green; and wax-free surface	No curd; direct flowering	Resistant to both *Xcc*15 and clubroot race 4	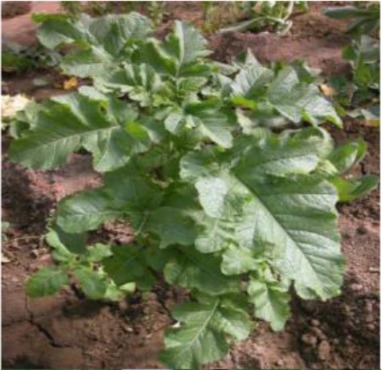
IL1	Grayish green; possesses auricles	Yellow and firm curd; short pedicel		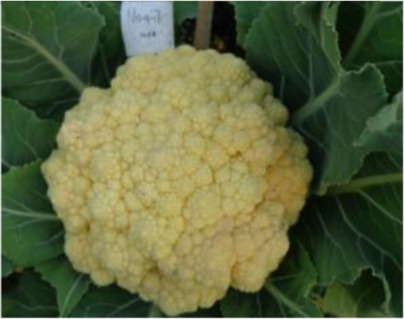
IL2	Pale grayish green; shallowly serrated margins; possesses auricles	White and firm curd; purple florets, short pedicel	Resistant to *Xcc*15	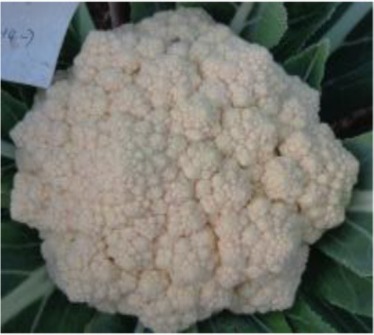
IL3	Green; serrated margins; no auricles	Pale and greenish curd; long pedicel	Early inflorescent; phosphorus content: 73 mg/100 g	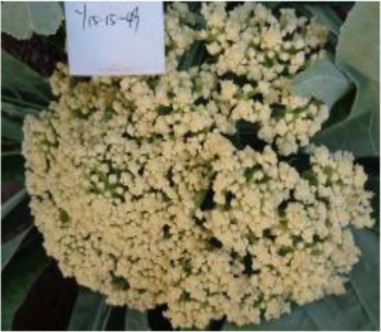
IL4	Light grayish green; serrated margins; no auricles	White and firm curd; short pedicel		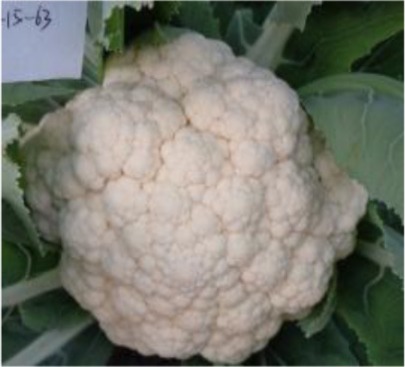
IL5	light green; wavy margin; possesses auricles	Pale green and soft curd; long pedicel		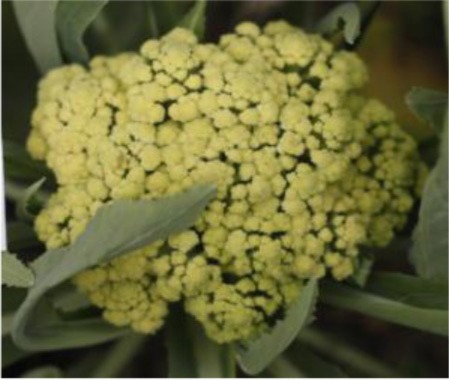
IL6	Pale grayish green; wavy margin; wing-like	White; firm, medium curd; white florets	Resistant to clubroot race 4	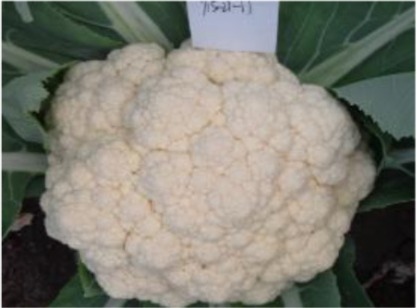
IL7	Pale grayish green; elongated; no auricles	No obvious curd; flower buds in clusters with leaf folder		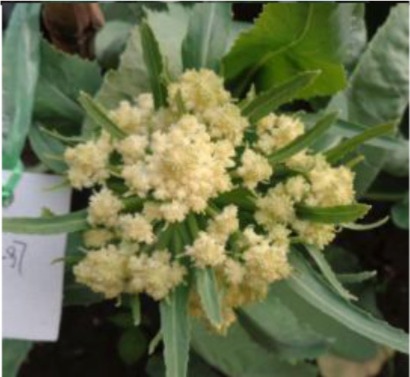
IL8	Light grayish green; serrated margins; no auricles	Pale yellow and scattered curd; long pedicel		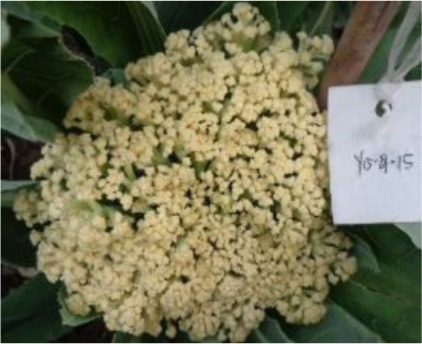
IL9	Green; serrated margins; possesses auricles	Yellowish and loose curd; long pedicel	Potassium content: 236 mg/100 g	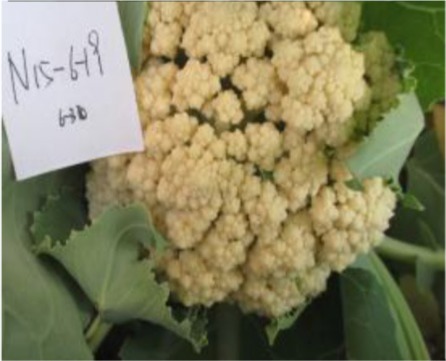
IL10	Pale grayish green; serrated margins; possesses auricles	Creamy white and loose curd; purple flower buds; long pedicel		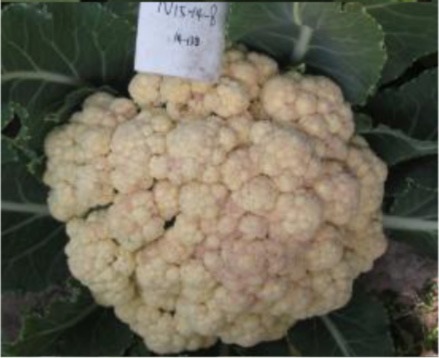
IL11	Similar to parent “Korso”	Creamy white and soft tomentose curd; short pedicel	Resistant to *Xcc*15	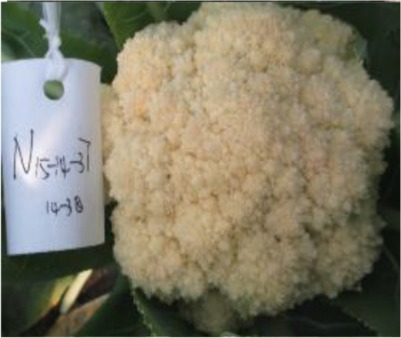
IL12	Pale grayish green; serrated margins; possesses auricles	Yellowish green and soft curd; purple flower buds; long pedicel	Early inflorescent	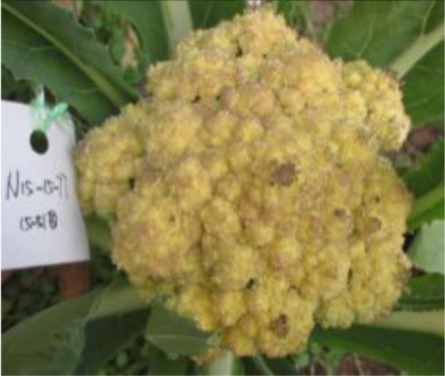

### Chromosomal constitution of the putative introgression lines

**Figure 3:**
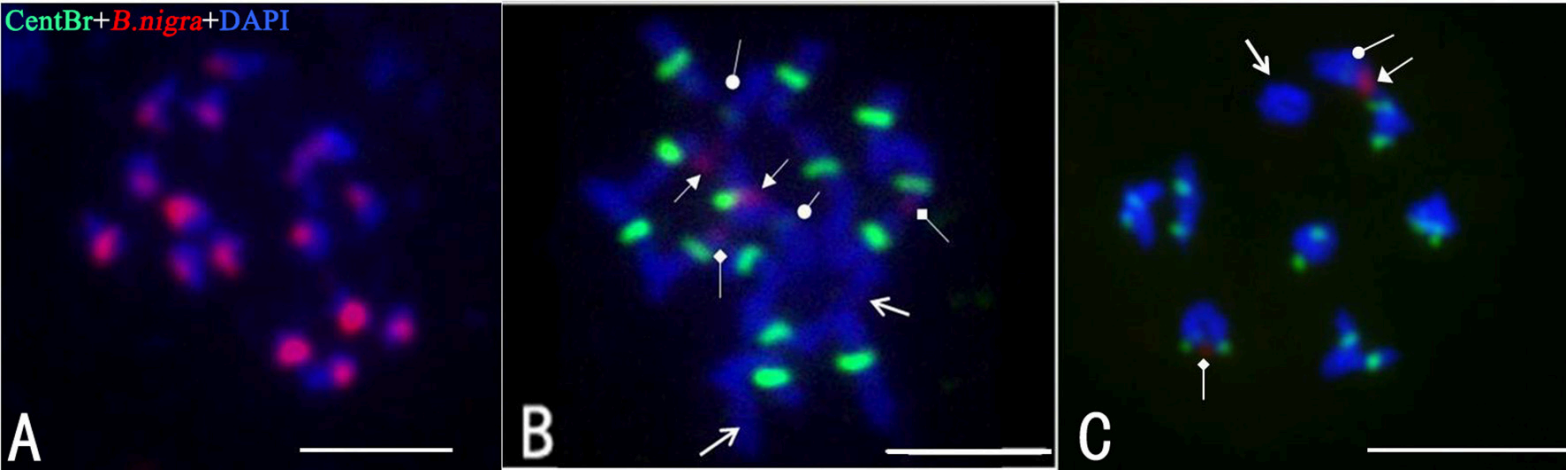
**Fluorescence ***in situ*** hybridization (FISH) analysis of the introgression lines using CentBr and “G1/1” genomic DNA as probes. (A)** Meiosis metaphase I of “G1/1.” **(B)** Mitosis metaphase I of “Korso.” **(C)** Meiosis prometaphase I of IL2. Triangle- and diamond-headed pointers indicate strong and weak red signals, respectively; circle-headed pointers indicate weak green signals; regular arrows indicate chromosomes with no signals. Bar = 5 μm.

Figure 3 shows the results of multi-color FISH on chromosomes of “G1/1,” “Korso,” and 28 putative ILs. The two parent lines exhibited different hybridization patterns with both probes. As expected, when using “G1/1” genomic DNA, all 16 “G1/1” chromosome pairs exhibited red signals (Figure 3A). In “Korso,” red signals (2 strong and 2 weak; Figure 3B) were detected on only two pairs of satellite chromosomes that likely correspond to the hybridization signals of 45SrDNA (Fukui et al., 1998).

No signals were detected on “G1/1” chromosomes using the CentBr probe. In contrast, seven pairs of strong signals and one pair of weak signals were detected on “Korso” chromosomes (Figure 3B); the latter was hybridized to chromosomes that also exhibited strong red signals. Only 1 “Korso” chromosome pair exhibited no signals (Figure 3B).

Most cells in the putative ILs contained 18 chromosomes and exhibited hybridization patterns identical to “Korso.” The results of genomic *in situ* hybridization (GISH) found no “G1/1” chromosomal segments present in these putative ILs, likely because GISH cannot detect introgressions from *Brassica* chromosomes due to their small size and compact structure.

### Genetic sequence variation analyzed using SSR and AFLP markers

We analyzed the sequence introgression and variation in 12 ILs (IL1–12) using 65 SSR and 77 AFLP markers that were polymorphic between the parents. The results of SSR and AFLP profiling revealed 1799 loci from both parents, including 682 (37.9%) those were polymorphic. The IL profiles were similar to the “Korso” profile, but loss of 5–13 “Korso”-specific bands were common. Additionally, all putative ILs were confirmed to possess anywhere from 3 to 33 “G1/1”-derived fragments. However, we noted that in 3 ILs, SSR markers failed to detect “G1/1”-derived bands but AFLP profiling revealed 20–25 “G1/1”-specific loci, indicating that the latter method is more effective when the introgression size is small. Next, all 12 ILs contained *B. carinata*-specific loci (35–49), and we were able to amplify 1–7 new bands from them.

These results clearly showed that all 12 lines were ILs and that genomic-sequence variations were common among them. Notably, IL genomes were not simply similar to the “Korso” genome but with some “G1/1”-derived fragments added. For example, we noted that some ILs lacked bands present in “Korso,” or possessed fragments lacking in “Korso.” Moreover, several *B. carinata*-specific bands were present in all ILs, hinting at the occurrence of common changes in the early stages of both somatic hybridization and naturally occurring hybridization.

**Figure 4:**
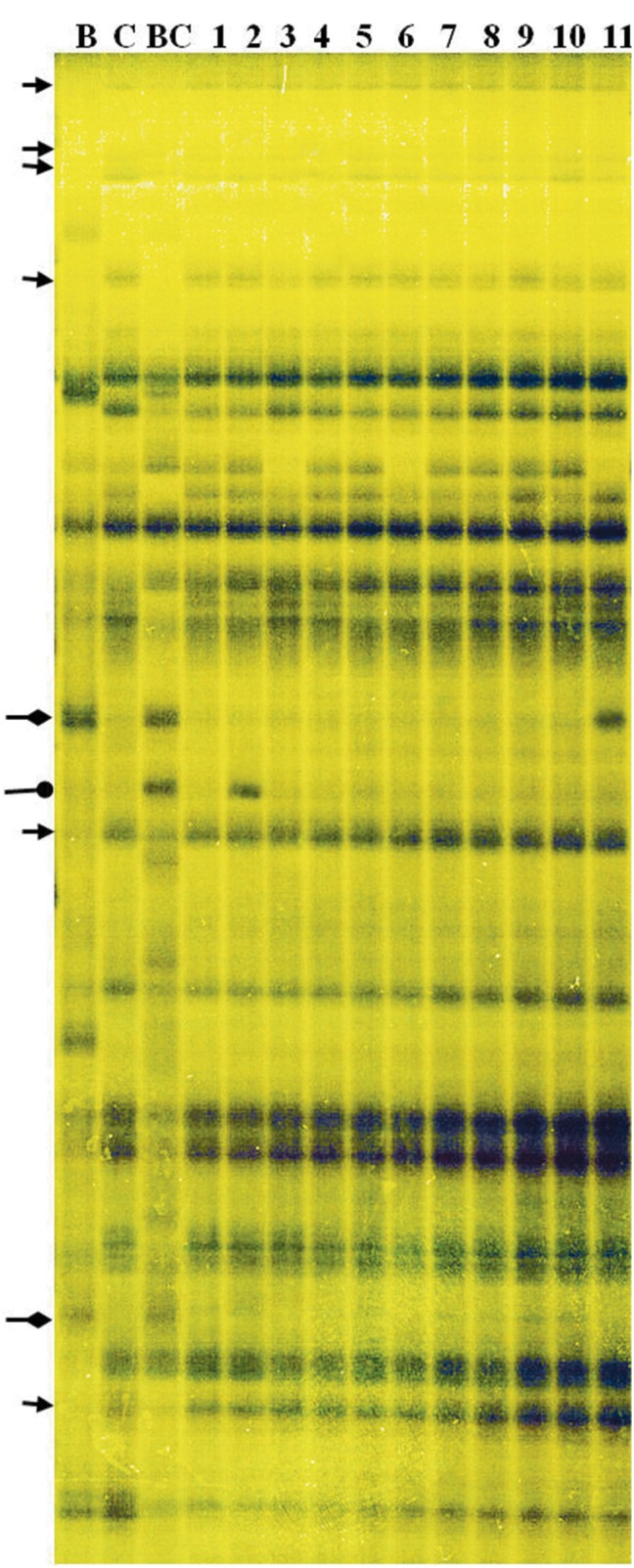
**Amplified fragment length polymorphism (AFLP) fingerprinting of the parents and ILs, with primer pair E45–M57**. C: “Korso,” B: “G1/1,” BC: *B. carinata*, 1–11: introgression lines. Arrows indicate “Korso”-specific bands, diamond-headed pointers indicate “G1/1”-specific bands, and circle-headed pointers indicate *B. carinata*-specific bands.

DNA profiling of the ILs revealed 1493 “Korso,” 1423 “G1/1,” and 1559 *B. carinata* fragments; 1117 of these were shared by both parents (Figure 4). The number of fragments present in the profiles of the 12 representative ILs ranged from 3 to 33, with an overall frequency of 7.5%, whereas the number of fragments lost ranged from 7 to 32, with an overall frequency of 5.1% (Table 2). Note that the percentages of “G1/1”-fragment presence and “Korso”-fragment loss were respectively based on the 306 “G1/1” fragments that did not co-migrate with “Korso” and the 376 “Korso” fragments that did not co-migrate with “G1/1.” In addition, the 12 ILs possessed 1–7 new fragments (1.4%) that were not present in the “Korso” profile, and 4–49 (15.5%) *B. carinata*-specific fragments from the 226 that did not co-migrate with “Korso” and “G1/1.”

**Table 2:** **DNA profiles and phenotypic summary of partial introgression lines (IL1-12)**.

**Introgression lines (ILs)**	**No. of *B. nigra*-specific bands and markers**	**Deletions in ILs**	**New bands in ILs**	***B. carinata*-specific bands and markers**	**Phenotype**
IL1	25: (23 AFLP), BrID10227, nia-m037a	12: (8 AFLP), BrID10205, nia-m037a, At3g55005a, cnu-m472a	7: (6 AFLP), BrID10223	40: (32 AFLP), BrID10205, BrID101161, BrID10223, At3g55005a, cnu-m472a, cnu-m052a	Leaf color and leaf type like the cauliflower; small, white, and firm flower head
IL2	26: (25 AFLP), BrID10227	13: (8 AFLP), BrID10205, BrID10381, BrID10173, nia-m037a	6 AFLP	35: (32 AFLP), BrID10985, BrID101161, BrID10381	Leaf color and leaf type like the cauliflower; small, white, and firm flower head
IL3	31: (29 AFLP), BrFLC2(R2), BrID10227	16: (9 AFLP), BrID10277, BrID10205, BrFLC2(R2), nia-m037a, cnu-m472a, nia-m063	6 AFLP	49: (41 AFLP), BrID10277, BrID10205, BrFLC2(R2), BrID101161, cnu-m472a, cnu-m052a	Leaf color and leaf type like the cauliflower; small, white, and firm flower head
IL4	20: (18 AFLP), BrFLC2(R2), BrID101081	23: (8 AFLP), BrID10277, BrID10205, BrFLC2(R2), BrID10881, BrID101157, BrID101183, BrID90311, BrID10493, BrID10223, BrID10173, nia-m037a, nia-m063a	6 AFLP	40: (32 AFLP), BrID10277, BrFLC2(R2), BrID90430, BrID101161, cnu-m052a	Leaf color and leaf type like the cauliflower; small, white, and firm flower head
IL5	22: (21 AFLP), At4g36960	26: (9 AFLP), BrID10277, BrID10205, BrID10881, BrID101157, BrID101161, BrID10971, BrID10381, BrID90311, BrID10493, BrID10173, nia-m037a, At3g55005a, nia-m063a, At3g51260	6 AFLP	40: (33 AFLP), BrID90430, BrID10381, BrID10493, At3g55005a, nia-m063a, cnu-m052a	Leaf color and leaf type like the cauliflower; loose, light yellow flower head; some light purple areas
IL6	27: (25 AFLP), BrID10227, At4g36960	11: (7 AFLP), BrID10205, BrID10493, cnu-m472a	6 AFLP	35: (31 AFLP), BrID10493, cnu-m472a, cnu-m052a	Leaf color and leaf type like the cauliflower; small, white, and firm flower head
IL7	32: (31 AFLP), At4g36960	30: (9 AFLP), BrID10277, BrID10205, BrID10881, BrID10381, BrID101183, BrID10971, BrID90311, BrID10103, BrID10493, BrID10101, BrID10275, BrID101199, nia-m037a, At3g55005a, cnu-m472a, At3g51260	6 AFLP	48: (40 AFLP), BrID10277, BrID90430, BrID101161, BrID10381, At3g55005a, cnu-m472a, cnu-m052a	Leaf color and leaf type like the cauliflower; white and firm flower head
IL8	27: (25 AFLP), BrID10985, BrFLC2(R2)	32: (13 AFLP), BrID10205, BrFLC2(R2), BrID101157, BrID101183, BrID90311, BrID10493, BrID10101, BrID10971, BrID10275, BrID101199, BrID10173, At1g58220, nia-m037a, At3g55005a, At4g36960, At3g51260	6 AFLP	49: (41 AFLP), BrID10277, BrID10985, BrID10205, BrFLC2(R2), BrID90430, At3g55005a, cnu-m052a	Waxy leaf and loose flower head
IL9	33: (31 AFLP), BrFLC2(R2), BrID10227	17: (11 AFLP), BrID10277, BrID10205, BrFLC2(R2), BrID101157, nia-m037a, cnu-m472a	6 AFLP	45: (38 AFLP), BrID10277, BrID10205, BrFLC2(R2), BrID101161, cnu-m472a, cnu-m052a	Loose flower head
IL10	25 AFLP	17: (8 AFLP), BrID10881, BrID101157, BrID101183, BrID10971, BrID10493, nia-m037a, At3g55005a, At3g51260	6 AFLP	34: (31 AFLP), BrID101161, At3g55005a, At3g51260	Waxy leaf and loose flower head
IL11	3: BrID10227, nia-m037a, At3g55005a	7: BrID10277, BrID10205, BrID101157, BrID10381, nia-m037a, At3g55005a, Ni4-B10	1: Ni4-B10	4: BrID10277, BrID10205, BrID10381	Ligule present; small, tight flower head
IL12	5: BrID10277, BrID10985, nia-m037a, At3g55005a, At2g38130	27: BrID10205, BrFLC2(R2), BrID10881, BrID101157, BrID101183, BrID90311, BrID10971, BrID10103, BrID10493, BrID10101, BrID10275, BrID101199, BrID10173, At1g58220, nia-m037a, At3g55005a, cnu-m472a, nia-m063a, Ni4-B10, At3g51260	2: BrID10223, Ni4-B10	5: BrID10277, BrID10223, BrID90430, At2g38130, nia-m063a	Gray-green leaf color; firm, small flower head
Average	23.0	19.3	5.3	35.3	
Frequency	7.5%	5.1%	1.4%	15.5%	

### Epigenetic changes in methylation patterns analyzed with MSAP

**Figure 5:**
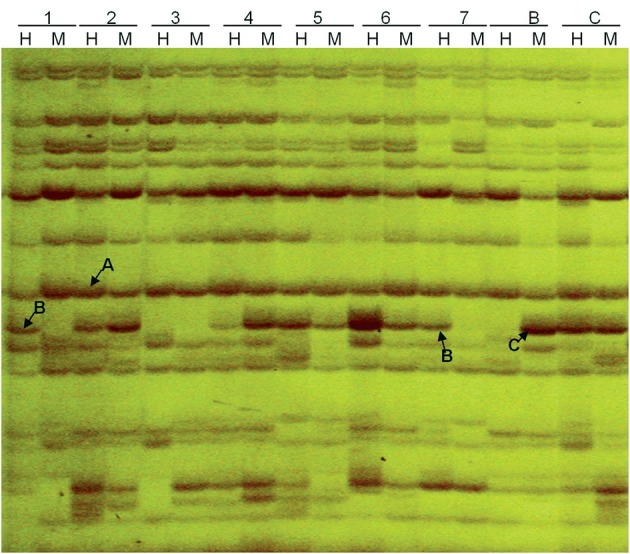
**Methylation-sensitive amplified polymorphism (MSAP) profiling based on primer combinations ETTC + HMTCC**. 1–7: introgression lines, B: “G1/1,” C: “Korso,” H: DNA cleaved with EcoRI + HpaII, M: DNA cleaved with EcoRI + MspI. A, B, and C indicate three separate band types.

The MSAP analysis used isoschizomers *Msp*I and *Hpa*II, which differ in their sensitivity to cytosine methylation (Shaked et al., 2001; Liu et al., 2015). Our comparison of the profiles generated from *Eco*RI-*Msp*I and *EcoR*I-*Hpa*II digestion revealed three kinds of band classifications (Figure 5). First, A type was characterized by fragments from both the *EcoR*I-*Msp*I and *Eco*RI-*Hpa*II digestions, representing non-methylated sites (or methylation within a single strand). Second, B type was characterized by half-methylation sites, present in *Eco*RI-*Hpa*II digestion but not in *Eco*RI-*Msp*I digestion. Third, C-type bands stemmed from complete methylation sites present in *Eco*RI-*Msp*I digestion but not in *Eco*RI-*Hpa*II digestion. Using 16 MSAP primer combinations amplifying clear and stable bands, we detected 401 fragments in parents, with 290 present in “Korso” but not in “G1/1.”

Table 3 summarizes variation in methylation patterns and their heredity in parents and the ILs. The average frequency of hypermethylated loci in different ILs was 12.4%, while the average frequency of hypomethylated loci was 4.8%.

**Table 3:** **Variation in cytosine methylation across the partial introgression lines (IL1-12)**.

**Introgression lines (ILs)**	**Hypermethylation in ILs**	**Frequency (%)**	**Hypomethylation in ILs**	**Frequency (%)**
IL1	42	14.5	7	2.4
IL2	36	12.4	9	3.1
IL3	35	12.1	8	2.8
IL4	39	13.5	22	7.6
IL5	28	9.7	18	6.2
IL6	32	11.0	17	5.9
IL7	38	13.1	12	4.1
IL8	28	9.7	10	3.5
IL9	34	11.7	8	2.8
IL10	42	14.5	22	7.6
IL11	40	13.8	20	6.9
IL12	36	12.4	13	4.5
Average	35.8	12.4	13.8	4.8

## Discussion

### Somatic hybridization and introgression can potentially increase genetic diversity

The low genetic diversity in modern Brassica varieties is concerning because it reduces potential genetic gains in breeding programs. To combat this problem, ILs containing fragments from related species can be used to generate improved cultivars. This method has proven successful in many crops, including wheat (Liu et al., 2007), rice (Rangel et al., 2008), potato (Chavez et al., 1988), eggplant (Mennella et al., 2010), *B. napus* (Primard-Brisset et al., 2005; Leflon et al., 2007), barley (Johnston et al., 2009), tomato (Menda et al., 2014), and rye grass (Roderick et al., 2003).

In this study, we generated a set of ILs using somatic hybridization between cauliflower and black mustard. The original objective was to select disease- and drought-resistant ILs for use in a Brassica breeding program. In fact, these ILs showed considerable genetic diversity in many morphological and physiological traits beyond resistance. Some of these traits are useful for increasing cauliflower adaptation to stressors. For instance, IL2 and IL6 respectively exhibited strong resistance to black rot and clubroot, major Brassica diseases with worldwide distribution. Other ILs exhibited commercially desirable traits such as early maturation and high nutritional quality, especially high phosphorus and potassium content (IL3 and IL9), as well as less desirable traits such as loose flower heads, Early inflorescent, and long pedicel. This diversity indicates that all of the ILs could be a source of new alleles for developing Brassica cultivars. Thus, somatic hybridization provides an effective and efficient means of achieving introgression into a crop species from its wild relatives and should continue to be important in producing novel germplasm for breeding programs.

### Combining cytological and molecular methods effectively identified brassica introgression lines

Although GISH has been successfully used to investigate genomic relationships in several agriculturally important genera, including Brassica (e.g., Lysak et al., 2005), and has detected introgressions in crops such as wheat (e.g., Liu et al., 2015), GISH was unable to detect black mustard chromosomal segments in our ILs. We believe this outcome was likely due to the small and compact Brassica chromosomes or else very small introgression sizes. We dealt with this issue by first using multi-color FISH to identify chromosome composition and then combining SSR and AFLP markers to detect “G1/1” sequence introgression. Primer sources and amplification results from the genomes of *B. rapa, B. nigra, B. oleracea*, and *B. carinata* are shown in Table S1. As those data indicate, the primers are usable in multiple Brassica species and can detect abundant polymorphism. Moreover, compared with the SSR primers, AFLP markers were more effective when working with small introgressions, such as those in our ILs (Table 2). In sum, our study demonstrated that Brassica ILs can be clearly identified with a combination of cytological and molecular methods.

### Genetic changes from somatic hybridization in the brassica introgression lines

The extensive phenotypic variation of the ILs was caused by genetic and epigenetic changes in the ILs relative to their parents. However, black mustard introgression was actually more limited (7.5%; Table 2) than other ILs, such as the somatic hybrids between wheat and tall wheatgrass (Liu et al., 2015). We believe the low introgression was probably due to suboptimal UV treatment. Thus, treatment factors such as irradiation time and intensity will require further testing and validation to achieve higher introgression rates. At the same time, the amount of introgression observed may be enough to achieve considerable genomic diversity. For example, in stable rice introgression lines derived from intergeneric hybridization (followed by successive selfing) between rice (*Oryza sativa* L.) and a wild relative (*Zizania latifolia* Griseb.), very low introgression (0.1%) by foreign DNA was enough to trigger up to 30% of the genomic changes (Wang et al., 2005). The mechanism underlying these changes is unclear, but some researchers have suggested that they happen very early, through a cryptic pathway that differs from conventional or unorthodox meiotic recombination of homeoalleles between rice and Zizania (Zhang et al., 2013).

Similar to previous findings (Wang et al., 2005), we also observed sequence loss and new bands (Table 2) following somatic hybridization, backcrossing, and self-pollination (Figure 1). The frequency of fragments lost (5.1%) was higher than presence of novel bands (1.4%). It is not entirely clear why the percentage of novel bands was so much lower, but one reason may be that some of them were *B. carinata*-specific, and *B. carinata*-specific fragments occurred at a relatively high frequency (15.5%). Thus, future investigations may need to confirm the exact nature of the lost and novel bands, as well as exclude any *B. carinata*-specific loci.

Our results were consistent with previous reports in artificial hybrid *B. carinata* (BBCC, *x* = 17), produced via crossing and polyploidization between *B. nigra* (BB, *x* = 8) and *B. oleracea* (CC, *x* = 9). Around 47% of the hybrid *B. carinata* (BBCC) genome possessed isoenzyme sites specific to natural allotetraploid *B. carinata* (Jourdan and Salazar, 1993). Given this similarity, we propose that the early generations of artificial synthesis and natural polyploid evolution of both genomes may have experienced the same change events. Supporting this idea, previous analyses of recombination in other genera, such as the homoploid hybrid sunflower (*Helianthus anomalus*), showed that newly synthesized hybrids converged on the linkage pattern of wild hybrids within five generations (Rieseberg et al., 1996).

Based on our results and those of previous studies, we propose that genetic changes in Brassica ILs occur via the following mechanisms. First, alien genetic elements are incorporated into cauliflower early in hybridization, through the activation of transposable elements or DNA methylation, as suggested in rice introgression lines (Wang et al., 2005). Next, chromosomal rearrangements occur through intergenomic translocations or transpositions (Udall et al., 2005) and homoeologous pairing (Leflon et al., 2006; Nicolas et al., 2007). During Brassica evolution, ancestral group rearrangements contributed to the high homology found especially in the three linkage groups of the B genome (B5, B6, and B4) and the A genome (A5, A6, and A4) (Panjabi et al., 2008). Finally, consecutive selection results in rapid sequence elimination, as reported in synthetic hybrids and hybrids less related to the original parents (Osborn et al., 2003; Pires et al., 2004; Gaeta et al., 2007; Zhang et al., 2013).

### Changes to DNA methylation in the brassica introgression lines

Cytosine methylation plays an important role in epigenetic gene regulation at both the transcriptional and the post-transcriptional levels (Paszkowski and Whitham, 2001). Our ILs exhibited fairly high proportions of change to methylation patterns (17.2%; Table 3), compared with several other plant hybrid/allopolyploid systems analyzed to date using MSAP. For instance, in resynthesized allotetraploid Arabidopsis suecica, 8.3% of the fragments experienced methylation changes (Madlung et al., 2002), whereas in newly synthesized allohexaploid wheat, ~13% of the loci saw alterations to cytosine methylation (Shaked et al., 2001). In contrast, a 23.6% change to cytosine methylation was observed in asymmetric somatic wheat introgressions (Liu et al., 2015). Taken together, these data allow us to conclude that somatic hybridization induced a broad spectrum of cytosine-methylation changes that perturbed gene expression to a larger extent than allopolyploidization.

Consistent with previous findings (Zhang et al., 2013), changes to hypermethylation (9.7–13.5%; Table 3) occurred more frequently than changes to hypomethylation (2.4–7.6%). Zhang et al. (2013) proposed that this pattern may be caused by the activation and subsequent silencing of some transposable elements after hybridization, but this hypothesis requires more data for confirmation. Additionally, the methylation patterns in ILs may be related to changes in enzymatic machinery, as well as the expression of siRNAs and long noncoding RNAs (Goll and Bestor, 2005; Wierzbicki et al., 2008; Zhang et al., 2013).

In conclusion, somatic hybridization mimics many of the genetic alterations induced by polyploidization or sexual wide hybridization, but to a stronger extent and with considerably less time. Therefore, somatic hybridization is both effective and efficient in achieving introgression of a crop species and its wild relatives. Moreover, this approach provides a potential means to explore the genetic and epigenetic events induced by “somatic genomic shock” (Liu et al., 2015).

## Author contributions

GW conceived the research work and wrote the paper. FL provided plant materials and intellectual advice on the project. JL performed molecular and FISH experiments. JZ and SH performed the field management and character survey. XZ performed the methylation-sensitive amplified polymorphism analysis. NG participated in the technical guidance of relative experiment. MZ and YZ performed materials classification and preservation. YW provided technical guidance for pathogen-resistance assays.

### Conflict of interest statement

The authors declare that the research was conducted in the absence of any commercial or financial relationships that could be construed as a potential conflict of interest.
