# Supplementary material for: Genetic and Epigenetic Alterations of Brassica nigra Introgression Lines from Somatic Hybridization: A Resource for Cauliflower Improvement
Source: Front Plant Sci. 2016 Aug 30;7:1258. doi: 10.3389/fpls.2016.01258 (PMC5003894; doi:10.3389/fpls.2016.01258)
Supplement: Supplementary file 1 [file Table1.DOC]

Supplemental Table S1-1. Polymorphic primers and amplification results

|  |  | Amplification results | | | |
| --- | --- | --- | --- | --- | --- |
| Name | Linkage groups | *B. rapa* | *B. nigra* | *B. oleracea* | *B. carinata* |
| BrID10277 | A1 | single band | many bands | many bands | many bands |
| BrID10985 | A1 | many bands | many bands | many bands | many bands |
| BrID10307 | A1 | many bands | no band | many bands | many bands |
| ENA28(R1) | A1 | many bands | many bands | many bands | many bands |
| BrFLC2(R2) | A2 | single band | single band | many bands | many bands |
| BrID10595 | A2 | many bands | many bands | many bands | many bands |
| BrID10199 | A2 | single band | many bands | single band | single band |
| BrID10205 | A2 | single band | no band | many bands | many bands |
| BrID10207 | A2 | many bands | many bands | many bands | many bands |
| BrID10759 | A2 | many bands | no band | many bands | many bands |
| BrID101197 | A2 | single band | no band | many bands | many bands |
| BrID90283 | A3 | many bands | many bands | many bands | many bands |
| BrID90039 | A3 | many bands | no band | many bands | many bands |
| BrID90430 | A3 | many bands | no band | many bands | many bands |
| BrID10041 | A3 | many bands | no band | many bands | many bands |
| BrID10703 | A3 | many bands | no band | many bands | many bands |
| BrID101081 | A3 | many bands | single band | many bands | many bands |
| BrID10881 | A4 | many bands | many bands | many bands | many bands |
| BrID10987 | A4 | many bands | no band | many bands | many bands |
| BrID90277 | A4 | many bands | no band | many bands | many bands |
| BrID10689 | A4 | many bands | no band | many bands | many bands |
| BrID90143 | A4 | many bands | no band | many bands | many bands |
| BrID10723 | A4 | many bands | many bands | no band | many bands |
| BrID10779 | A4 | many bands | no band | many bands | many bands |
| 8C0522 | A4 | single band | single band | many bands | many bands |
| BrID10857 | A5 | many bands | no band | many bands | many bands |
| BrID90333 | A5 | many bands | no band | many bands | many bands |
| BrID101183 | A5 | single band | no band | single band | single band |
| BrID101063 | A5 | many bands | no band | many bands | many bands |
| BrID10383 | A5 | single band | no band | single band | single band |
| BrID10971 | A5 | many bands | many bands | single band | many bands |
| BrID90105 | A6 | many bands | single band | no band | single band |
| BrID101157 | A6 | many bands | single band | many bands | many bands |
| BrID101161 | A6 | many bands | no band | single band | many bands |
| BrID10381 | A6 | many bands | no band | single band | many bands |
| BrID90311 | A6 | single band | no band | many bands | single band |
| BrID10869 | A7 | many bands | no band | many bands | many bands |
| BrID10497 | A7 | many bands | no band | many bands | many bands |
| BrID10493 | A7 | many bands | no band | many bands | many bands |
| BrID10101 | A7 | single band | no band | many bands | many bands |
| BrID10103 | A7 | many bands | no band | many bands | many bands |
| BrID10275 | A8 | single band | no band | single band | single band |
| BrID10933 | A8 | many bands | no band | many bands | many bands |
| BrID101199 | A8 | many bands | no band | single band | single band |
| BrID10727 | A8 | single band | no band | single band | many bands |
| BrID10173 | A9 | many bands | many bands | single band | many bands |
| BrID10955 | A9 | many bands | no band | many bands | many bands |
| BrID10227 | A10 | many bands | single band | no band | many bands |
| BrID10223 | A10 | many bands | no band | many bands | many bands |
| cnu-m371a | A3 | single band | no band | single band | single band |
| nia-m014a | A5 | single band | no band | single band | many bands |
| cnu-m472a | A5 | no band | no band | single band | single band |
| nia-m037a | A6 | many bands | many bands | many bands | single band |
| nia-m063a | A7 | single band | single band | single band | many bands |
| cnu-m052a | A7 | single band | no band | single band | single band |
| Ni4-B10 | N11 | no band | single band | single band | single band |
| Ni4-C1 | N15 | no band | single band | no band | single band |
| Ni2-H06 | ? | no band | single band | no band | single band |
| At1g58220 | B1, G7, A1 | single band | no band | many bands | many bands |
| At1g03180 | B7, G3, A8 | single band | single band | single band | single band |
| At1g68310 | B2, G5, A2, A7 | single band | single band | single band | single band |
| At2g38130 | B4, G6 | many bands | single band | single band | many bands |
| At3g55005a | B3, G8, A7, B4 | single band | single band | single band | many bands |
| At3g51260 | B8, G2, A9 | many bands | many bands | many bands | many bands |
| At4g36960 | B2, G5, A3 | single band | single band | single band | single band |

Notes: Under “Linkage groups,” A: *B. rapa*, G: *B. nigra*, B: B-genome in *B. juncea*, N: *B. napus*, ?: unknown; under “Name,” cnu & nia: A-genome SSR markers, N: B-genome SSR markers, At: intron polymorphism (IP) markers from *B. juncea*.

**Supplemental Table S1-2. Sequences of amplified fragment length polymorphism (AFLP)** adapters and primers

| Adapters and primers | Nucleotide sequence 5'—3' | Adapters and primers | Nucleotide sequence 5'—3' |
| --- | --- | --- | --- |
| *Eco*R I adapter | CTCGTAGACTGCGTACC  AATTGGTACGCAGTC | *Msp* I adapter | GACGATGAGTCCTGAG  TACTCAGGACTCAT |
| E0A | GACTGCGTACCAATTCA | M0C | GATGAGTCCTGAGTAAC |
| E32 | GACTGCGTACCAATTCAAC | M47 | GATGAGTCCTGAGTAACAA |
| E33 | GACTGCGTACCAATTCAAG | M48 | GATGAGTCCTGAGTAACAC |
| E35 | GACTGCGTACCAATTCACA | M49 | GATGAGTCCTGAGTAACAG |
| E36 | GACTGCGTACCAATTCACC | M50 | GATGAGTCCTGAGTAACAT |
| E37 | GACTGCGTACCAATTCACG | M53 | GATGAGTCCTGAGTAACCG |
| E38 | GACTGCGTACCAATTCACT | M54 | GATGAGTCCTGAGTAACCT |
| E40 | GACTGCGTACCAATTCAGC | M57 | GATGAGTCCTGAGTAACGG |
| E41 | GACTGCGTACCAATTCAGG | M58 | GATGAGTCCTGAGTAACGT |
| E44 | GACTGCGTACCAATTCATC | M59 | GATGAGTCCTGAGTAACTA |
| E45 | GACTGCGTACCAATTCATG | M60 | GATGAGTCCTGAGTAACTC |
|  |  | M61 | GATGAGTCCTGAGTAACTG |
|  |  | M62 | GATGAGTCCTGAGTAACTT |

Supplemental Table S1-3. Sequences of methylation-sensitive amplified polymorphism (MSAP) adapters and primers

| Adapters | Nucleotide sequence 5'—3' |
| --- | --- |
| *EcoR* I-adapters I | CTCGTAGACTGCGTACC |
| *EcoR* I-adapters II | AATTGGTACGCAGTC |
| *Hpa* II/*Msp* I-adapters I | GATCATGAGTCCTGCT |
| *Hpa* II/*Msp* I-adapters I | CGAGCAGGACTCATGA |
| Preselective primers | Nucleotide sequence 5'—3' |
| *EcoR* I + A | GACTGCGTACCAATTCA |
| *Hpa* II/*Msp* I + 0 | ATCATGAGTCCTGCTCGGT |
| Selective primer combinations used in MSAP (5’→3’) | |
| *EcoR* I-AAC + *Hpa* II/*Msp* I-TCA | *EcoR* I-ACA + *Hpa* II/*Msp* I-ATA |
| *EcoR* I-AAC + Hpa II/*Msp* I-ATA | *EcoR* I-ACA + *Hpa* II/*Msp* I-ACT |
| *EcoR* I-AAC + *Hpa* II/*Msp* I-ACT | *EcoR* I-ACA + *Hpa* II/*Ms*p I-CAT |
| *EcoR* I-AAC + *Hpa* II/*Msp* I-CAT | *EcoR* I-AAC + Hpa II/*Msp* I-TCA |
| *EcoR* I-AAG + *Hpa* II/*Msp* I-TCA | *EcoR* I-ACT + *Hpa* II/*Msp* I-ATA |
| *EcoR* I-AAG + *Hpa* II/*Msp* I-ATA | *EcoR* I-ACT + *Hpa* II/*Msp* I-ACT |
| *EcoR* I-AAG + *Hpa* II/*Msp* I-ACT | *EcoR* I-ACT + *Hpa* II/*Msp* I-CAT |
| *EcoR* I-AAG + *Hpa* II/*Msp* I-CAT | *EcoR* I-ACA + *Hpa* II/*Ms*p I-TCA |
